# Communication for collaborative computation: two major transitions in human evolution

**DOI:** 10.1098/rstb.2021.0404

**Published:** 2023-03-13

**Authors:** Daniel Dor

**Affiliations:** Department of Communication, Tel Aviv University, Tel Aviv 69978, Israel

**Keywords:** major evolutionary transitions, human evolution, language, mimesis, collaborative computation, instructive communication

## Abstract

This paper presents and defends the following theoretical arguments: (1) The uniqueness of the human condition lies in the fact that only humans engage in *collaborative computation*, where different individuals work together on shared computational challenges. Collaborative computation is the foundation of our cumulative cultures. (2) Collaborative computation requires individuals to engage in *instructive communication*, where senders do not just send messages to receivers—but also send them instructions that the receivers are obliged to follow in the course of computing the messages. (3) The process of human evolution was driven throughout by the invention and development of tools of instructive communication. (4) In this process, two separate major transitions should be identified. The first was made possible by the toolkit of representational gestures (pointing, eye contact, manual demonstration, pantomime and more) that Merlin Donald called the toolkit of *mimesis*. Mimesis allows for collaborative computation as long as the information requiring computation is available for direct experiencing by the participants. The second was made possible by *language*, the tool that allowed its users, for the first time, to engage in collaborative computations of information they did not experience together—through the systematic instruction of the mental computations of imagination.

This article is part of the theme issue ‘Human socio-cultural evolution in light of evolutionary transitions’.

## 1. Introduction: human evolution and its transitions

The discourse on human evolution is deeply complicated by the simple fact that we are still struggling to pin down the essence of our uniqueness as a biological species. As opposed to other defining properties of biological entities, which are well understood and thus present the theory of evolution with well-defined objects for investigation, the question of what it is that defines us—what it is that defines the human condition—is still open.

For a very long while, the dominant view of the human condition and its uniqueness concentrated on the individual human mind (or soul, or brain, or central nervous system). It identified our uniqueness in two cognitive capacities that have always been thought of as lacking in the other animals: the capacity for sophisticated thought and the capacity for language. The two capacities were thought of as inherent properties of human minds: God-given until quite recently, genetically given in the last century. These innate capacities defined the space of possibility for human knowledge, communication and action, and formed the universal foundation upon which our uniquely cumulative cultures emerged and developed. Noam Chomsky's theory of language as a formal tool for the representation of thought was the most influential exposition of this view in the second half of the twentieth century [1–4]. According to the theory, the essential characteristic of language is its supposedly universally shared infinitely generative grammar: the capacity for such grammar is a foundational, genetically given property of the human mind.

This view of language provided the basis for Maynard-Smith and Szathmáry's account of their last major transition: the emergence of grammatical language, ‘with its unlimited hereditary potential … opened up the possibility of open-ended cumulative cultural evolution’ [5,6(p. 10110 for quotation),^7^]. This statement should be read within the overall context of the theory: the major transitions in the evolution of life are those moments in which higher levels of organization emerge, and biological units, which exist up to that moment as independent individuals, are incorporated within the new level of organization—and adapt themselves accordingly. What happens in these moments is a transition in *individuality*: the higher-level entity is from now on the individual under evolutionary selection. Such transitions, moreover, require a supporting transition in the tools of *information transfer*, to allow the stable inheritance of the new level of organization. Maynard-Smith and Szathmáry's statement thus says that: (i) the transition to the human condition was a transition in individuality: communities based on cumulative culture (partially) replaced individuals as the units of selections; and (ii) this transition was supported by the emergence of language as the tool that stabilized the transfer of culture between generations.

Today, we are witnessing a paradigm shift in our understanding of the human condition. We no longer think of cumulative culture as a result of our nature. Cumulative culture *is* our nature: we are a cultural species. The ‘secret of our success', as Joseph Henrich puts it, lies not in ‘our individual brainpower’, but in the ‘collective brains of our communities' [8, p. 6]. This view, to be sure, is much closer in spirit to Maynard-Smith and Szathmáry's overall conception: it thinks about the human condition as inherently collective.

Different authors portray the new paradigm in different ways, but the emergent consensus is wide enough. It weaves together five insights:

(i) Individuals in the other species are ‘individual intentional agents' [9,10]: the contents of their mental lives are mostly private and solitary (not always: this will become important soon). Our mental lives, on the other hand, are overwhelmingly collective: we live by sets of collective beliefs and norms, pools of collective knowledge—Cecilia Heyes calls them *gadgets* [11]—that developed through the long histories of our communities, and are stored, so to speak, *on the Net*—at the social level referred to by different authors as the collective brain [8], the intersubjective level [12], or the levels of joint and shared intentionality [9,10]. Language, within this picture, is not an innate capacity but a gadget: a collectively constructed communication technology [13].
(ii) Contrary to the old belief, our performance on a variety of cognitive tasks is often weaker than that of the apes [14]. Where we are incomparably superior, however, is in tasks involving social learning and mind reading—which are exactly the capacities required for a life of *downloading* from the Net [15]. We learn what we need to know, including our languages, from the others around us. The sophistication of our thoughts is due to the fact that we build them on the accumulated wisdom of past generations.
(iii) Human communities show levels of cooperation, information sharing and division of labour, accompanied by collective norms of behaviour, all of which are unattested in our relatives [16,17]. The collective nature of our practical engagements with the world, and the cultural nature of our collective brains, are two sides of the same coin. Together, they define us as a species.
(iv) The other animals are essentially solitary intentional agents, but not always. Various components of our unique evolutionary dynamics already appear, in more rudimentary forms, in some of the other animals, especially the apes. Social learning is ubiquitous in the animals [18]; the apes are very clearly capable of mind reading, at least to a certain extent [19,20]; individuals in many other species cooperate with each other [21] and play together [22]; almost all species communicate, and many species share information about the world, mainly in the form of alarm calls and food calls [23]; the socially aware brain is much older than us [24]; many species show clear signs of local cultures, which are transmitted from one generation to the next through social learning [25]. This deep continuity allows for the explanation of the *origin* of the human condition in terms of a process that begins with gradual, local and quantitative advancements in everything that we already inherited from our ape-like past, and reaches its peak when all these advancements, taken together, cross a certain threshold—the veritable Rubicon—and let us enter the human condition.
(v) The story of our evolution after we crossed the Rubicon is essentially about a long process of gene–culture coevolution [8,13,26–29]. Human cultures and human individuals (including their genetic makeup) evolved together, in coevolutionary spirals: communities gradually morphed into collective units, and continued to accumulate cultural knowledge and deepen their dependency on cooperation. Individuals gradually came to be selected for their ability to participate in the collective game: to understand the others, communicate with them, learn from them and cooperate with them. This selection resulted in a rich set of partial accommodations at all the relevant levels: behavioural, cognitive, emotional, developmental and genetic.

What all this means, and this is the essence of the paradigm shift, is that the causal relationship between the properties of our minds (or brains) and the properties of our cultures is reversed: our minds are the way they are mainly *because* we adapted ourselves (individually) to the requirements of our collective-cumulative cultures. This will be the starting point for my discussion: it is a huge step forward in our understanding of ourselves.

I do believe, however, that this paradigm shift still leaves the most important issue out of the picture. It conceptualizes the Rubicon as a *quantitative* threshold: on both banks of the Rubicon, biological creatures cooperate, learn from each other, warn each other of dangers, and manoeuvre their way around in complex social realities—the difference is that on their bank all this only happens in rudimentary fashion, whereas it takes over as the organizing principle of life on our bank. All this is probably correct as such, but it cannot be the whole story. There was a very clear *quality* to the Rubicon: something very deep happened when our ancestors crossed it.

## 2. The transition in individuality: collaborative computation

To get closer to the qualitative nature of the Rubicon, let us adopt the explanatory strategy developed by Simona Ginsburg & Eva Jablonka in their search for the essence of an earlier transition—the transition to consciousness [30]. Following Tibor Gánti [31], they suggest that we search among the many components involved in a transition and look for an *evolutionary marker*: a component of the dynamic that clearly marks the *completion* of the transition. Instead of looking at our Rubicon from its past (standing, so to speak, with the animals, on its ancient bank), we should look at it from its future: when can we be certain that we're already standing firmly on the human bank? To be sure, we do not want to walk further away from the bank: we are looking for the first, minimal indication of completion.

This change of perspective makes it immediately clear that most of the central components of the transition—social learning, information sharing, cooperation, mind reading, intersubjectivity and culture—cannot possibly be its evolutionary markers, for the simple reason that we already see them in the animals. We are a cultural species, but we are not *the* cultural species. We are the only species with cumulative culture, but this—according to everybody—is only an indication that we have crossed a certain threshold in terms of our capacities (individual and collective). So, what can we do that may be considered a minimal indication of completion? Here's a partial list:

(i) While social learning undoubtedly played a central role in our story, we only know that the transition has been completed when we encounter *explicit teaching*, in which teacher and pupil work together, dialogically, to meet the challenges of learning. Other animals do sometimes behave in ways that make learning easier (catch prey and release it for pups, for example) but they do not engage in explicit teaching [32,33]. When we see A learning from B, we cannot be sure where we are. When we see B teaching A, and A giving feedback to B, we can.
(ii) While practical cooperation, doing things together, was as important as social learning in the story, the marker only shows itself in the *negotiated coordination* of the collective effort [10]. In the animals, A and B can hunt together, for example, and coordinate their moves by individualistically taking into account the moves of the other. We know that the transition is complete when we see A and B deciding together on the goal and strategy for the hunt, revising their decision together as the hunt proceeds, and figuring out together, once the hunt is over, what they need to learn from it for the next time.
(iii) Mind reading is another crucial component of the story, but when we see A and B reading some of each other's mental contents, we cannot be sure. The apes can do that. When we see A and B communicating about their mental contents—presenting them to each other, asking each other about them, arguing about them, trying to find common ground—that is when we know the Rubicon has been crossed.
(iv) Information sharing is important, but it cannot be thought of as the marker. Animals share information. We can only know the transition has been completed when we see A and B *constructing* information together.

What is the common denominator here? Well, the above list calls on us to turn our attention from the mental contents to the processes of their production. On both sides of the Rubicon, we see collective mental contents. Only on the human bank, however, do we see collective contents that are themselves collectively *produced*. The production of contents becomes an interactive affair: different individuals work together on shared mental challenges.

In terms of the contents of cognition, as we have seen, other animals are not always solitary: for some contents, they rely on the others. They are, however, always solitary in terms of the *production* of their mental contents. Every individual produces all its mental contents by itself. The solitary nature of animal cognition is *computational*. At any given moment, the entire chain of computation—from the allocation of attention, through perception and detection, the analysis of the perceived data, the on-going processes of learning and the construction of memory, the dynamics of problem-solving and prediction-making, all the way to the decision-making and its implementation in practice—takes place separately and autonomously inside the central nervous system of each individual.

The crucial thing to see here is that solitary computation allows quite a lot to be achieved collectively. When A and B read each other's minds, the chains of computation in their minds are autonomous, but the mutual reading has a collective effect. The same is true when A learns from B: the chains of computation are separate, but skills and capacities are transferred between generations. It is true when A and B separately figure out how to behave towards the other: their autonomous decisions create a social scene. And it is also true when A and B share information about the world. Consider alarm calls: A detects a threat, emits the call and sets out to defend itself; B hears the call, analyses it and sets out to defend itself. The call and its effect are public, but the chains of computation are still separate and autonomous.

On our side of the Rubicon, things are qualitatively different. A's and B's nervous systems sometimes still work as autonomous computational devices, but they very often assume the role of nodes on a network of what we may call *collaborative computation*. Their individual chains of computation are woven together by messages sent back and forth by both sides. They recruit each other for collaborative computational efforts, define their computational goals together, dissect and split the computational efforts between them, compute partial results, send them to each other, compare and evaluate them, go through rounds of dialogical discussion, and eventually produce collective contents that emerge from their negotiations and arguments—contents that surpass whatever each of them could produce autonomously.

The most important property of our networks of collaborative computation may be highlighted by a brief comparison with the networks of *distributed computation* currently in use in the computer world. In these networks, multiple computers connect and communicate, dissect computational tasks and split these tasks between them. In this sense, collaborative computation is a form of distributed computation. But distributed computation, at least in its current stage of development, is hopelessly inflexible in comparison with our networks: computational tasks are pre-determined, and so are the divisions of labour, the server–client relationships, and the communication protocols.

Networks of collaborative computation are emergent, plastic phenomena: they emerge, change and disappear based on need and capacity. Individuals maintain partial computational autonomy, recruit each other for tasks of computational collaboration, constantly change leadership positions and update divisions of labour, and engage in a dialogue that surpasses the mere distribution of the computational effort: different individuals, with different (and often conflicting) perspectives on the same problem*, argue* about the best way to define the problem and approach it. The computational tasks are not pre-determined: they are discussed and defined in collaboration.

All this allows for the collaborative capacity that stands at the root of our cumulative cultures: the capacity for collaborative *innovation*. Animals sometimes invent too, but only individually [34]. We are the only species in which different individuals invent together. Collaborative computation allows various individuals, each of whom brings to the table a different set of skills and perspectives, to make partial dialogical contributions to a collaborative effort of innovation, in which solutions are found that could not be found by any of the participants alone.

This, I would like to suggest, is the qualitative marker of the transition to the human condition: the emergence of collaborative computation. It was a transition in computational individuality. The uniqueness of our cultures—the fact that they are innovation-based and cumulative, and the fact that they rely on shared values, norms and identities—should be attributed to this revolutionary change. Social learning, cooperation and mind reading are not enough: they are still solitary capacities. To stabilize a way of life based on the inheritance and further development of the collective wisdom of past generations, our ancestors had to begin to connect their nervous systems on networks of collaborative computation.

We usually think about the collective capacities of the apes as the precursors to ours—which they definitely are—but now we can think about them in another way: as the highest collective achievements possible as long as brains are computationally solitary. Brains appeared in the Cambrian as autonomous computational devices [30]. From that moment on, animals developed (among other things) higher and higher levels of sociality, with their affordances and complexities, and tools of communication designed for manoeuvring in social space; various forms of cooperation developed and stabilized; social learning began to allow the construction of rudimentary cultures. Eventually, apes developed the capacity to make educated guesses about some of the mental dynamics of the others. This, quite simply, is the furthest one can get without collaborative computation.

Solitary computation severely constrains the envelope of sociality. When our ancestors crossed the Rubicon, they did not just push their collective efforts beyond a quantitative threshold: they broke the glass ceiling of solitary computation, and opened a totally new space of collective possibilities (and collective problems) that is uniquely our own. In a very real sense, the entire evolutionary history of the human species, from the very beginning until today, is the story of this new space – how we gradually filled it up and how it gradually changed us.

## 3. The transition in information transfer: instructive communication

The question we may now ask is this: if the story is indeed about computational individuality, a transition from solitary to collaborative computation, what type of upgrade in information transfer does such a transition require?

We have characterized the animals as computationally solitary, and this applies just as well to their communication. At any given moment in the course of a communicative exchange, where A and B take their turns as senders and receivers, the entire chains of computation involved in their respective roles take place separately and autonomously inside the nervous system of each of them. Everything that A goes through, from the decision to transfer some information (let us call it the message M) to B, to the implementation of the decision in actual behaviour (barking, for example), is independent from what B goes through, from the detection of the bark through the interpretation of M to the decision to react (say, by barking back), and *vice versa*. This computational independence of senders and receivers seems to be obvious—how can it be otherwise? —but the fact of the matter is that collaborative computation requires something completely different.

For A to engage B in a project of collaborative computation, A has to do more than just transfer some information to B. A also has to convey to B what A expects B to do with the information: to *instruct* B in the process of the computation of the information. B, on the other side of the exchange, has to do more than just receive the information from A and interpret it at will: B has to comply with A's instructions. It is easy to see that without this additional layer of communication, the collaborative project would be impossible. Let me call this type of communication, which is specifically designed for collaborative computation, *instructive* communication.

In solitary communication, A behaves in a way that sends a message M to B, and B analyses the behaviour independently and receives M. In instructive communication, on the other hand, A behaves in a way that sends B *two* messages: the message M, and a set of instructions for mental computation that B has to comply with in the process of computing M.

What this means is that the computational dynamics inside B's nervous system, when B plays the role of the receiver, *are no longer fully independent*. B allows A to send a long hand, so to speak, into the computational dynamics taking place inside B's nervous system, and instruct them in their work. B actually relinquishes control of some of his or her computations, and allows A to partially manage them.

This, I would like to suggest, is the communicative foundation of the human condition. The animals live in solitary networks in which they take turns in sending independent messages to each other. We do that too, but what makes us unique is the fact that we take turns in doing something radically different: instructing each other's minds in the course of the effort of collaborative computation. This is how our ancestors managed to stabilize a way of communal life based on collaborative computation: they gradually developed and accumulated—throughout human evolution (and then human history)—an ever-growing variety of unique communicative tools that are specifically designed for instructive communication.

While we humans share many communicative behaviours with the animals, and especially with the apes, we also use a wide set of communicative behaviours and tools that are uniquely our own. What makes all of them our own is the fact that they are (at least partially) designed for instructive communication. Language is obviously the most prominent among our communicative tools: in [13] I show that it is best analysed as a tool of instructive communication, and we shall get back to this shortly. But language is not alone. On the one hand, we have everything that humans have developed *after* language, in the last 100 thousand years or so—from plastic art, through writing, all the way to the social media. While these deserve their own re-analysis as tools of instructive communication, I will not touch on this issue here.

On the other side of language, we have an entire toolkit of communicative behaviours that are already uniquely human but also very clearly *pre-linguistic*. A huge amount of scientific attention has been given in the last few decades to this toolkit [35–42]: only humans use intentional gestures (manual, bodily, facial and vocal) to direct each other's attention to things or away from them; to demonstrate and teach manual and bodily skills; to ‘describe’ physical things without words; to depict events (through mimicry and pantomime); to express agreement or disagreement, understanding or confusion, and ask for discursive repair; to ask for information and negotiate collective decisions; to organize and manage practical collaborative projects; and to construct and maintain social cohesion and social identity (in ritual, theatre, music and dance). Only humans use eye-contact to seal agreement, and blinking to send feedback.

The skills and capacities involved in these pre-linguistic behaviours begin to emerge in children long before language, and when children begin to acquire language they very clearly do it on the basis of intensive communication with their caregivers and peers, where the entire toolkit is used. In the other animals, on the other hand, the toolkit is completely absent. The apes show that they can learn to use some of it, in a very rudimentary way, only in the artificial environment of the laboratory, where they are exposed to human behaviours and encouraged to adopt them [43]. They learn to point, for example, but only at food that they cannot reach by themselves, and only to a human who they think can help. In the wild, they show no signs of pointing. The impressive linguistic achievements of the apes in the laboratory are based on their pointing achievements [44], and testify more than anything else to their plasticity in learning. In the wild, where there is no pointing, there is no language.

Merlin Donald was the first in modern times to identify this toolkit as the missing link between ape culture and language-based human culture [35]. He characterized the entire set of behaviours as instances of what he called *mimesis* (not to be confused with mimicry). In mimetic behaviours, according to Donald's definition, the body is intentionally used as a representational device (mimicry is simply one such behaviour). Here, again, I believe that the definition deserves an update: mimetic behaviours are indeed intentional usages of the body as a representational device, but this as such is not what makes them so important. The key to mimesis lies elsewhere: it is the first toolkit that our ancestors developed that is designed for instructive communication.

Consider pointing. What is it that happens when A points at a certain object for B? The usual answer is that A sends some perceptual information to B: M is the object pointed at. This is of course correct, but it only tells half of the story. What happens in this event, which eventually allows B to get the message, is a process in which A instructs B's visual computations: A tells B *where to look*. The instruction says: look at me; then look at my arm and concentrate on my finger; follow the line from my finger to the ground; see the object there. When B follows these instructions, he or she actually allows A to take command, if only momentarily, of his or her perceptual behaviour. For the duration of the pointing event, A handles B in a way that is similar to the way we handle a camera, the way we move it around to put the lens in line with the object we want to capture. B follows the instructions, and sends instructive messages back, by eye-contact, nodding and gesturing: I saw it, you can stop pointing; or, I see nothing there, can you point more accurately? Eventually, they look each other in the eye to seal the agreement. What happens here is not just information sharing: it is a sophisticated instructive dialogue.

The same thing happens when A explicitly teaches B a manual skill, such as tying a complex knot with a rope [45]. The message is the actual technique, but everything that characterizes the *demonstration*—the slow motion, the dissection of the procedure into stages, each of which is shown separately, the accompanying gestures and facial expressions designed to attract attention to this facet or the other of the process, the demonstration of mistakes that should be avoided—all these are designed to instruct B in the computational process of learning the technique. Here, too, B continuously sends instructive messages back to A: I did not get the second stage, can you do it more slowly? Can you show it from the other side? All the tools in the toolkit of mimesis include components that are designed for instructive communication. Together, they allow groups of individuals to do what the other animals cannot: to teach, to demonstrate, to plan collaborative projects, to negotiate and coordinate them in real time, to re-enact them later in order to draw conclusions for next time, to stabilize practical divisions of labour, and to construct a collective sense of identity and common purpose.

What makes all these tools members of the same toolkit is the fact they allow communicators to instruct the same subset of their interlocutors' mental computations: the mental computations involved in their *perceptual experiencing* of the immediate environment around them. Mimesis thus allows for full-fledged collaborative computation of information that the individuals involved experience together. As a matter of fact, as long as the information is available for perception by all sides, mimesis is far superior to language as a tool of instruction.

Exactly because of this, however, mimesis is severely constrained by the condition of direct experiencing: it can do very little when the information is not presentable. Mimesis breaks the glass ceiling of *solitary* experiencing, but remains inside the realm of *experiencing* as such.

This is where language comes into the story. As I show in detail in [13], it is specifically designed by cultural evolution for instructive interactions dealing with information that is not available for experiential presentation. While mimesis instructs the senses, language instructs the *imagination*.

In a nutshell: language allows speakers to intentionally and systematically instruct their interlocutors in the process of imagining the intended content—instead of experiencing it. A provides B with a code, a structured list of the basic coordinates of the information—which B is then expected to decipher and use as a formal set of instructions for imagining. The interlocutor analyses the code, uses the words (or more technically, the morphemes) to raise past experiences from his or her own memory, and then reconstructs and recombines them, based on the grammatical arrangement of the morphemes, to produce a novel, imagined representation. Language allows communicators to instruct their interlocutors’ imaginary computations, to make them imagine things they have not experienced by themselves. This is what makes language unique: it breaks the glass ceiling of experience.

With language, groups of human individuals can engage in collaborative projects that are no longer limited to the here-and-now of their shared experiences: to share information gathered away from the group (this is Charles Hockett's *displacement* [46]), to create cultural worldviews that are based on stories from distant places and distant times time [47], and to populate their worlds with things that some of their members (and sometimes all of them) have never experienced but only *heard* about (often from someone who had also only heard about it). As importantly, language allows individuals to channel into each other's imagination new types of thoughts and ideas, requests and orders, questions and promises—exactly the types that are very hard, and often impossible, to express with mimesis.

Language brings about an explosion in the ability of the group to engage in collaborative computation: to work together on problems and solve them; to construct and maintain complex collaborative practical projects; to develop and teach new skills (those that cannot be taught with mimesis); to maintain a communal order that is partially based on imagination, because different members of the group live very different lives (this is the root of Benedict Anderson's ‘imagined communities' [48]); and to negotiate a collective worldview that now includes at its centre imagined entities, imagined beliefs (beliefs not based on experiencing, private and collective) and imagined norms—and everything else that follows from the new ability to instruct the computational dynamics of the others' imaginations.

All this, I would like to argue, allows for a major re-thinking of the process of human evolution, as a coevolutionary spiral driven throughout by the emergence and further development of tools of instructive communication: mimetic communication, then language (then the modern tools, which deserve their separate discussion). Each of these, in its turn, allows for the emergence and further development of new levels of collaborative computation, which allow the emergence and further development of new levels of cumulative culture. Throughout the process, individuals gradually come to be selected for their ability to participate in the game, in terms of both computation and communication, which means that every generation can push the entire system a bit further. Every such push deepens the dependency of communities and individuals on collaborative computation, which requires the invention of more tools of instructive communication, and so on and so forth.

## 4. Two major transitions: mimesis and language

Maynard-Smith and Szathmáry conceptualize their last major transition in terms of the co-emergence of cumulative culture and grammatical language. As they fully acknowledge, however, this merging of the emergence of language with the emergence of cumulative culture is quite problematic for a very simple reason: cumulative culture began to emerge a very long time before language. Following Chomsky and others, Maynard-Smith and Szathmáry estimate that language emerged in *Homo* *sapiens*, sometime within the last 200 thousand years. According to other estimates, language emerged around a half a million years ago, before *Homo* *sapiens* [49]. I think there are very solid reasons to accept the second view, but this way or the other no-one claims that language emerged much earlier than that.

The fact, however, is that we begin to see serious signs of the uniquely human evolutionary trajectory—where cultures begin to assume their cumulative nature—as far back as two million years ago, and all the signs are definitely there a million years ago or so: complex tool manufacturing [50], collective foraging and big game hunting [9,51], alloparenting [52], control of fire and cooking [53], and collective rituals [54]. All these definitely required a major upgrade in communication. Maynard-Smith and Szathmáry acknowledge the gap between the early beginnings and the emergence of language and put the burden of bridging this gap on what they call, following some of the linguistic literature, *protolanguage*: a system of communication with a small lexicon and no grammar. They make it clear that the period of protolanguage is not itself a transition: it is a preparatory stage on the way to the linguistic transition.

The notion of protolanguage is problematic for a wide array of reasons [55]. It emerged on the basis of the Generative definition of language itself, and as such it refers not to a first prototype of language (like the brothers' Wright proto-airplane), but to something that is decidedly not yet a language: it does not have a grammar. Maynard-Smith and Szathmáry assume that the capacity for protolanguage is shared with the apes, and suggest that it was recruited when hominins began to collectivize as a result of the dire need to communicate for survival [7]. In this scenario, protolanguage definitely looks like the Wright airplane: a first prototype of the thing itself.

This way or the other, the idea that something like protolanguage was the tool of communication responsible for our ancestors' collective achievements before full-fledged language misses out on the most important empirical fact about protolanguage. Wherever we see small lexicons emerge without grammar—in the beginning of language acquisition in children, or in rudimentary pidgin languages constructed for commerce between speakers of different languages—they clearly emerge on the basis of a very intensive engagement in mimetic communication. As a matter of fact, mimetic communication is incomparably more efficient than protolanguage as a tool for the on-site coordination of practical collaborative work—exactly of the type that challenged early *Homo* *erectus*. Even today, with our full-fledged languages, we very often retreat to mimesis when we do practical things together. When we teach complex practical skills—like tying complex knots, playing an instrument or practising martial arts—mimesis does most of the work: teaching these without mimesis, using only words, is virtually impossible.

All this strongly implies that the system of communication responsible for the achievements of pre-linguistic humans was mimesis, not protolanguage. There is no doubt that small lexicons without grammar played a role in the evolution of language: like the proto-airplane, they were probably the first viable prototypes of the new tool. They already did what language does—instruct the imagination—and when grammar emerged it allowed the same function to be served in a much more efficient way. But for such first prototypes of language to emerge, human communities must have already been thoroughly mimetic.

For Maynard-Smith and Szathmáry, as we have seen, the protolanguage period was a preparatory stage for the linguistic transition. Having replaced protolanguage with mimesis, we now face a dilemma: how do we conceptualize the mimetic period? Is it still preparatory, or is it a transition in and of itself? Following Donald, I will suggest that the mimetic period should be thought of as a full-fledged transition.

When our ancestors (most probably early *Homo* *erectus*) began to experiment with mimesis, they were already mature solitary experiencers: they already learned from each other, read each other's minds, communicated their way through social life, and maintained rudimentary cultures. Why did they begin to explore mimetic communication? Probably because they found themselves in circumstances in which solitary computation was no longer enough. In [13], I characterize these as circumstances of *epistemic dependency*: (i) A experiences something that calls for action, but cannot perform the action by himself or herself; (ii) B is in a position to act, but has not experienced what A has; and (iii) the survival of both depends on A's ability to make B understand what needs to be done. How did they manage to do it? We will never know: maybe they were individually smarter than the other apes, or more empathic or more communicative; maybe they had already managed to push their solitary cultures beyond that of the apes; maybe they were just lucky.

As the toolkit of mimesis stabilized, our ancestors found themselves on the other side of the Rubicon. They began to instruct each other's perceptual computations: to point at things for each other, to demonstrate, to teach, to look each other in the eye, to approve or disapprove. Gradually, communities began to map their experiential worlds together, and develop a collective, normative worldview. They could now begin to accumulate cultural accomplishments, and they gradually learned how to do so systematically and intentionally. On top of the earlier, ape-like social order, a new order emerged, based on prestige and knowledge, communicative skills and creativity. Individuals began to see that the others do not necessarily see them as they see themselves. New socially oriented emotions appeared: pride on the one hand, shame and guilt on the other. Individuals gradually adapted themselves to the new game.

By around half a million years ago, hominin communities (probably late *Homo* *erectus*) were already fully mimetic. Their survival depended on the collective implementation of complex collaborative projects, and these depended on the overall capacity of the community to compute together through mimetic communication. Major divisions of labour, most importantly between hunters and gatherers, where already stabilized. Individuals made various contributions to the collective effort, but their own survival already depended on the others doing their parts as well. After a million and half years of selection, children could in all probability already acquire mimetic skills, and enter the world of collaborative computation, without training: the relevant capacities were already (partially) genetically accommodated.

The invention of mimesis, in other words, did much more than prepare the ground for language. Mimesis *is* the tool of instruction that allowed our ancestors to cross the Rubicon of solitary computation and stabilize a way of life based on collaborative computation. This is an important piece of news: our age-old belief that the key to our uniqueness is language is wrong.

It was in this context, deep inside the human condition, that our fully mimetic ancestors began to experiment with language. Here, again, they may they have been smarter, more empathic and more trusting, and they may have reached a level of mimetic instruction that simply allowed them to make the extra step. In all probability, they were again forced into their explorations by epistemic dependency: mimetic instruction was no longer enough. This way or the other, they crossed the second Rubicon.

In [13], I show that this perspective allows for a natural (and obviously speculative) characterization of the evolutionary dynamics that brought us from these first experiments to our full-fledged languages. The central point is that it allows us to re-think the moment of *origin*. Instead of thinking about it as the moment in which language was born, we may now think about it as the moment in which the *function* of language was born. Well versed in mimetic instruction, the inventors of language may have begun by simply using the old tools of mimesis for the instruction of imagination. This new usage is easy to demonstrate. Suppose that A and B walk around looking for something. Then A sees it, looks at B and points at the thing. If B then follows the instruction, sees the thing and looks back into A's eyes with a nod of approval, we have a successful event of mimetic instruction. If B fails to notice anything where A points, the event fails. There is, however, another option: B may decide to trust A, realize that A does see something that he or she does not (because B's line of vision is blocked by a tree, for example), and act on this realization. What happens in this scenario is that B imagines the thing to be there, based on A's pointing: pointing is used for the instruction of imagination.

This re-thinking of the origin allows for a detailed, speculative characterization of the evolutionary process that ensued, as a process in which language is gradually extracted from its mimetic foundations, and gradually takes its own form—in a constant collective effort to increase the levels of success in the instruction of imagination. The tool is gradually built for the function, not the other way around. In the beginning, this dynamic shows itself in the emergence of the phonetic sound system from within the sound system of mimetic vocalization, and, more importantly for our purpose here, in the emergence of first lexicons. These essentially give names to everything that has already become part of the mimetically constructed world of collective experiencing. Every such name is an instructor: ‘this particular thing that we now experience together, next time I use this name imagine it’. This is how we still begin the journey of language acquisition: with mimetic instruction, most often pointing, and naming. The small lexicons allowed their users to instruct each other's imagination about what they did not experience together *in terms of what they did*.

The lexicons grew larger and more complex, and speakers began to concatenate signs, like pearls on strings, still without grammar. This was *protolanguage*: the first viable prototype of language. As speakers learned to concatenate longer and longer strings, a new type of problem emerged: the longer the string is, the more difficult it is to interpret correctly. The collective effort to minimize misunderstandings began to produce grammatical rules and constraints of different types, designed to mark the way towards the correct interpretation—and thus minimize ambiguity, vagueness and other sources of misinterpretation. Grammars emerged as collective solutions to a problem of communication and allowed the packaging of longer and longer strings in ways that kept them interpretable.

Throughout this evolutionary process, as communities deepened their dependency on the instruction of imagination, individuals were gradually selected for their capacity to participate. The two main capacities that emerged for language were the capacity for *fast speech* on the one hand and the capacity for *creative imagination* on the other. Like the other individualistic capacities, we find first signs of *basic* imagination in the animals [56,57]. It stands to reason that in the mimetic period individuals had already enhanced their imaginative capacities, but it was only with language, and for language, that imagination assumed its place at the centre of our cognitive uniqueness.

From all this, *Homo sapiens* emerged as a species whose entire existence depends on collaborative computation on the two levels, mimetic and linguistic, experiential and imaginative. As testimony to the two transitions, the instructive division of labour between mimesis and language is still with us, and so is the highly complex dialectic relationship that the two maintain between them: the ways language influences our experiences and the ways our experiences influence language. Children show us the same thing: they are born with an innate capacity for creative imagination, with a physiology already suited for fast speech, and with an innate desire to instruct and be instructed. They do not need help in learning how to point, demonstrate, mimic or speak.

## Data Availability

This article has no additional data.
